# Atypical Presentation of Parsonage-Turner Syndrome

**DOI:** 10.7759/cureus.8892

**Published:** 2020-06-28

**Authors:** Ramzi Ibrahim, Michael Krivitsky, Monica Nicola, Christopher C Zarour

**Affiliations:** 1 Internal Medicine, St. Joseph Mercy Oakland Hospital, Pontiac, USA; 2 Radiology, St. Joseph Mercy Hospital, Pontiac, USA

**Keywords:** parsonage turner syndrome, brachial plexopathy

## Abstract

Parsonage-Turner syndrome, also known as idiopathic brachial neuritis, is a multifocal, complex inflammatory disorder of the brachial plexus. It is an under-recognized diagnosis that requires increased vigilance to diagnose. We present a case of an abnormal presentation of Parsonage-Turner syndrome 10 weeks after a rotator cuff tear repair surgery. To diagnose this condition, other potential causes should be ruled out effectively with modalities such as magnetic resonance imaging (MRI) of the cervical spine and shoulder, electromyography (EMG), or recent history of inciting events.

## Introduction

Parsonage-Turner syndrome is an under-recognized syndrome with an extensive multifocal process. It has been hypothesized to be an autoimmune-mediated, although it is still primarily unknown [1]. It usually occurs after an inciting event, which may be exercise-induced, surgeries, recent vaccinations, or infections [2,3]. In general, the infectious cause is the most common inciting event, which accounts for 25-55% of cases. The infectious agent can be parvovirus B19, Epstein-Barr virus, or human immunodeficiency virus (HIV) [4]. The incidence of this syndrome is about two to three individuals per every 100,000 people, most commonly between the 3rd and 7th decades of life. This syndrome has also shown a greater predilection for males rather than females [5].

Historically, the classical form of this syndrome presents with excruciating pain, anytime up to a few weeks following an inciting event, with subsequent localized weakness or hypoesthesia/paresthesia in certain areas innervated by affected nerves. However, motor deficits are more common than sensory involvement [6, 7]. Although not fully understood, the distribution of neurological deficit fluctuates between patients. We herein present a patient that developed acute proximal arm weakness without pain 10 weeks after corrective rotator cuff tear surgery.

## Case presentation

A 60-year-old patient presented with right upper extremity weakness 10 weeks after a rotator cuff tear repair surgery. The postoperative course was uncomplicated. However, 10 weeks postoperatively, the patient woke up with sudden onset right upper extremity weakness. Deficits were seen mostly with active arm flexion, external rotation, and abduction.

On physical examination, there was notable weakness in the right upper extremity motor strength when compared to the left upper extremity, graded a 3/5 on the right and 5/5 on the left. Abduction was limited to 90 degrees on the right side and there was a significant weakness of the right biceps and right brachioradialis muscles. Reflexes were absent on the right biceps and brachioradialis muscles. The remainder of the physical exam was unremarkable.

**Figure 1:**
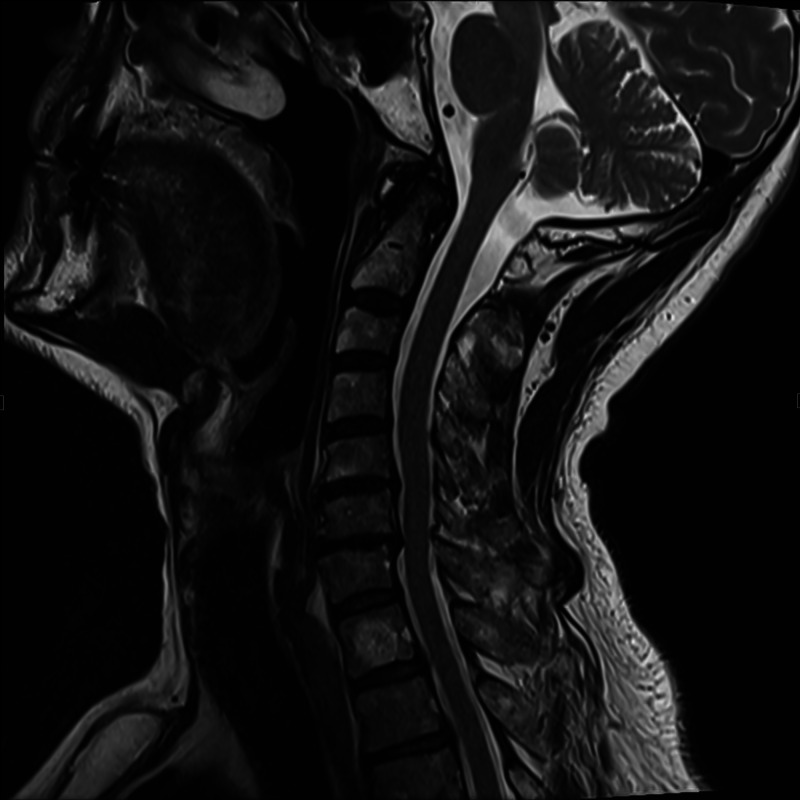
Sagittal MR Cervical Spine

**Figure 2:**
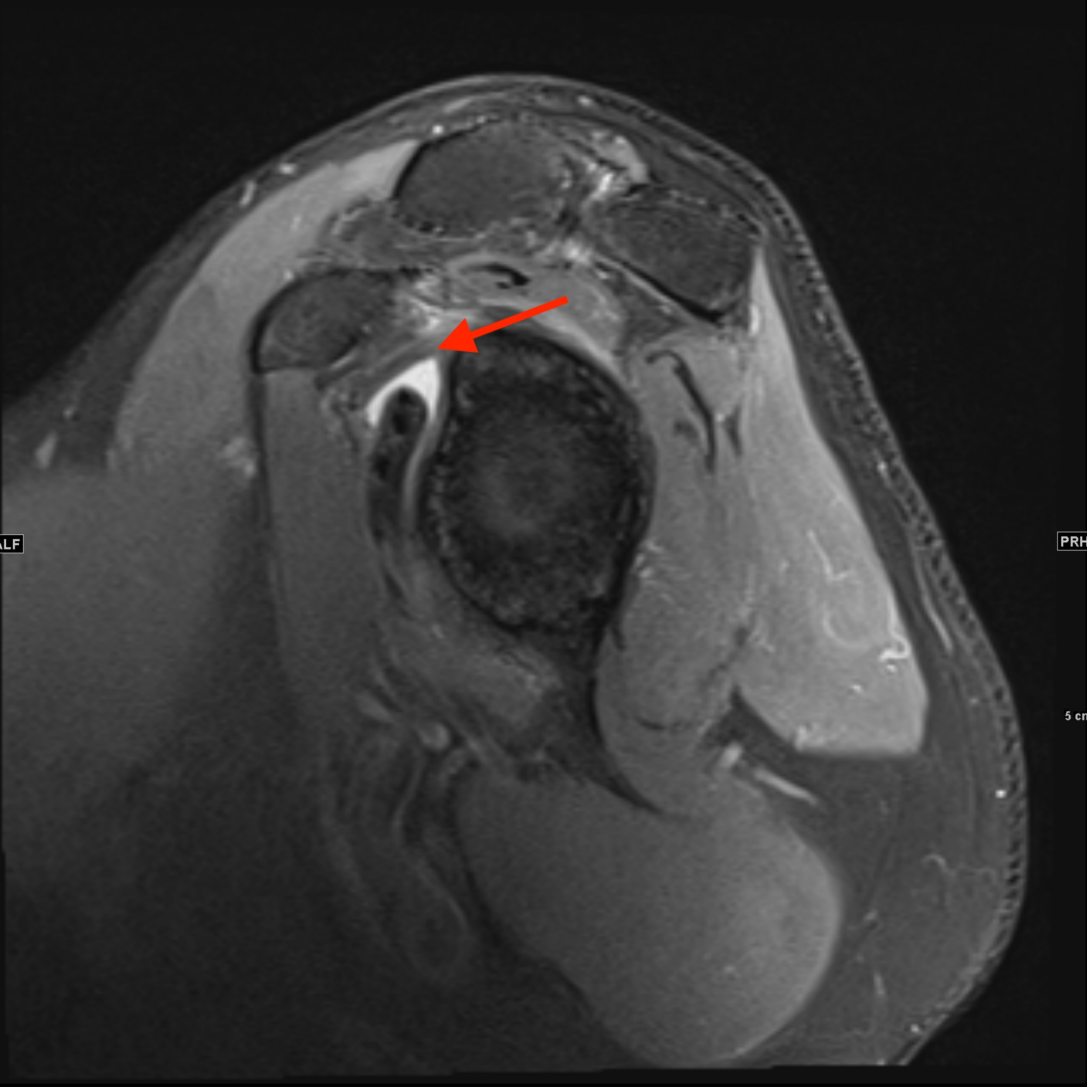
Sagittal MR Shoulder

**Figure 3:**
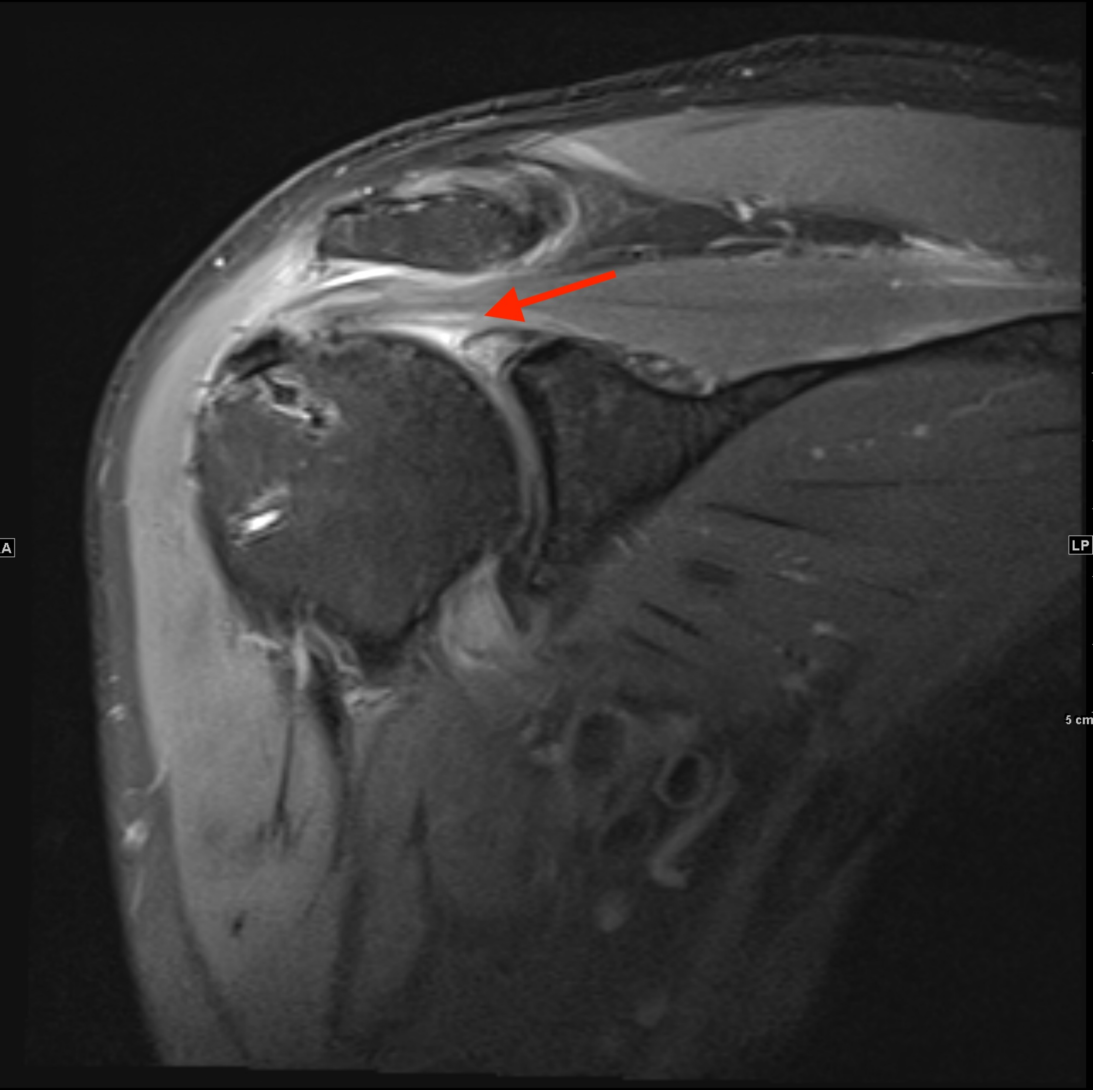
Coronal MR Shoulder

The initial laboratory workup was completely unremarkable. Therefore, imaging was ordered to determine the possibility of other similarly presenting diagnoses such as radiculopathy. Magnetic resonance imaging (MRI) of the cervical spine (Figure 1) was done which showed findings consistent with osteoarthritic changes, otherwise unchanged from a cervical spine MRI done two years ago. MRI of the right shoulder (Figures 2, 3) showed mild residual edema along the tendons of the rotator cuff muscles, mild amount of reactive fluid in the subacromial subdeltoid bursa, partial tears of glenoid labrum, and a mild glenohumeral joint effusion. Electromyography (EMG) study was significant for denervation of the biceps, deltoid, supraspinatus, and infraspinatus muscles with no involvement of the cervical paraspinal muscles. These findings suggest the involvement of C5-C6, localizing to the upper trunk of the brachial plexus.

Over the course of a few weeks, the motor function improved with an extensive course of physical therapy without pharmacological intervention. Parsonage-Turner syndrome was diagnosed with the support of clinical findings and EMG findings.

## Discussion

Parsonage-Turner syndrome is not a common diagnosis in clinical practice. It has been historically known as acute brachial neuritis, brachial plexopathy, or neuralgic amyotrophy. Unfortunately, this diagnosis is often confused among a variety of other similarly presenting disorders such as cervical spondylosis, adhesive capsulitis, cervical radiculopathy, and acute calcific tendinitis. It most commonly occurs following inciting events [2-3, 8]. Although not fully understood, the hypothesized pathophysiology is an autoimmune phenomenon with possible genetic predisposition and environmental contributors.

The classical presentation typically manifests within the first few weeks of inciting events and is characterized by pain and motor weakness. However, abnormal presentations may occur and do not follow the classical progression of symptoms [1]. In our patient, the late onset of symptoms, which occurred ten weeks after the inciting event, and the immediate motor weakness and the lack of pain, is an atypical manifestation of this syndrome. Diversity in symptoms is dependent on the affected nerves. For example, a patient may have only one or multiple nerves affected. In our patient, C5 and C6 were both involved and showed signs of denervation. Importantly, there should be attentiveness for the involvement of certain nerve roots that may have complicating outcomes such as phrenic nerve paralysis with resulting diaphragm dysfunction and breathing difficulties. To our knowledge, phrenic nerve involvement has been identified in about 7% of cases of Parsonage-Turner syndrome [3, 9].

The diagnosis of this condition involves adequate clinical findings supported by EMG results. Other diagnoses that may present in a similar manner should also be ruled out. Our patient’s physical examination findings were supported by findings seen on EMG, which showed denervation in the right-sided biceps, deltoid, supraspinatus, and infraspinatus muscles, which are coinciding with the involvement of the upper trunk of the brachial plexus. MRI of the cervical spine can be used to rule out cervical radiculopathy or mass effects.

The management is usually conservative. Physical therapy has been shown to alleviate and preserve the range of motion but does not improve recovery time. Medications have no role in management. The phenotypic variations of this syndrome among the population contribute to the heterogeneity in recovery time. For some, it may take up to three years for complete recovery, and some patients may continue to have residual deficits [10].

## Conclusions

The diagnosis of Parsonage-Turner syndrome can be challenging. The approach to diagnosis includes history, physical examination, and EMG of the affected muscle groups. Acknowledging the symptom variability allows for enhanced recognition and superior outcomes of patient care.
